# Supplementary material for: The association of maternal pre-pregnancy body mass index with macrosomia: a birth cohort study from China
Source: PeerJ. 2025 Nov 10;13:e20332. doi: 10.7717/peerj.20332 (PMC12614098; doi:10.7717/peerj.20332)
Supplement: Supplemental Information 2 [file peerj-13-20332-s002.docx]

**The association of maternal pre-pregnancy body mass index with macrosomia: a birth cohort study from China**

Mingxin Yan ^1,2†^, Yunbo Zhang ^2†^, Doudou Zhao ^3^, Yan Zhao ^4^, Danmeng Liu ^3^, Li Shan ^3^, Yang Mi ^3^, Leilei Pei ^2,*^ and Pengfei Qu ^3,5**^

^1^ Institute for Hygiene of Ordnance Industry, Xi'an 710065, China

^2^ Department of Epidemiology and Biostatistics, School of Public Health, Xi'an Jiaotong University Health Science Center, Xi'an 710061, China

^3^ Translational Medicine Center Northwest Women's and Children's Hospital, Xi'an 710061, China

^4^ The First Affiliated Hospital of Xi'an Jiaotong University Dermatology, Xi'an 710061, China

^5^ Central Laboratory, Beijing Obstetrics and Gynecology Hospital, Capital Medical University, Chaoyang, Beijing 100026, China.

*Corresponding author: Department of Epidemiology and Biostatistics, School of Public Health, Xi'an Jiaotong University Health Science Center, Xi'an 710061, China.

**Corresponding author: Translational Medicine Center Northwest Women's and Children's Hospital, Xi'an 710061,China.

E-mail addresses: [pll_paper@126.com](mailto:pll_paper@126.com) (L. Pei), xinxi3057@163.com (P. Qu).

†These authors have contributed equally to this work

**Supplementary Table 1** Association between pre-pregnancy BMI and three subgroups macrosomia according to logistic regression analysis.

| Variable | Grade 1 macrosomia | Grade 2 macrosomia | Grade 3 macrosomia |
| --- | --- | --- | --- |
|  | *Adjusted OR* (*95%CI*), *P* | *Adjusted OR* (*95%CI*), *P* | *Adjusted OR* (*95%CI*), *P* |
| Pre-pregnancy BMI |  |  |  |
| Under weight | 0.57(0.42~0.76), <0.001 | 0.42(0.13~1.39), 0.154 | 0.27(0.04~2.04), 0.203 |
| Normal weight | 1.00 | 1.00 | 1.00 |
| Overweight | 1.62(1.31~2.01), <0.001 | 2.80(1.47~5.32), 0.002 | 0.70(0.16~3.10), 0.637 |
| Obesity | 1.55(1.02~2.35), 0.041 | 3.46(1.19~10.10), 0.023 | 1.61(0.21~12.45), 0.647 |
| *P* for trend | <0.001 | <0.001 | 0.104 |

Adjusted for maternal age, education level, ethnicity, family financial situation, drinking before or during pregnancy, passive smoke before or during pregnancy, cold/fever before or during pregnancy, folic acid supplementation before or during pregnancy, parity, current GDM, fetal sex.

**Supplementary Table 2** The association between pre-pregnancy BMI and macrosomia in subgroups.

| Variable | *OR* (*95%CI*) ^a^ | | | |
| --- | --- | --- | --- | --- |
|  | Under weight | Normal weight | Overweight | Obesity |
| Maternal age, years |  |  |  |  |
| ≤24 | 0.48(0.21~1.13) | 1.00 | 0.93(0.37~2.34) | 0.69(0.09~5.40) |
| 25~29 | 0.69(0.48~0.99) | 1.00 | 2.00(1.47~2.71) | 2.04(1.15~3.63) |
| 30~34 | 0.47(0.27~0.82) | 1.00 | 1.66(1.21~2.28) | 1.58(0.87~2.89) |
| ≥35 | -^b^ | 1.00 | 1.67(0.88~3.18) | 2.16(0.62~7.56) |
| Educational level |  |  |  |  |
| Below high school | 0.17(0.04~0.72) | 1.00 | 1.23(0.68~2.22) | 2.00(0.85~4.70) |
| College/university | 0.64(0.42~0.88) | 1.00 | 1.92(1.52~2.43) | 1.84(1.16~2.91) |
| Postgraduate | 0.57(0.28~1.17) | 1.00 | 1.46(0.84~2.53) | 0.87(0.20~3.76) |
| Family financial situation |  |  |  |  |
| Poor | 0.60(0.25~1.46) | 1.00 | 1.19(0.62~2.28) | 2.71(1.27~5.77) |
| Moderate | 0.61(0.43~0.86) | 1.00 | 1.86(1.47~2.36) | 1.56(0.94~2.57) |
| Rich | 0.46(0.24~0.88) | 1.00 | 1.79(1.10~2.91) | 1.14(0.35~3.76) |
| Parity |  |  |  |  |
| Nulliparous | 0.62(0.45~0.85) | 1.00 | 1.66(1.30~2.13) | 1.67(1.03~2.69) |
| Multiparous | 0.40(0.19~0.83) | 1.00 | 1.92(1.35~2.72) | 1.95(1.01~3.77) |
| Current GDM |  |  |  |  |
| Yes | 0.56(0.40~0.76) | 1.00 | 1.65(1.28~2.14) | 1.45(0.84~2.49) |
| No | 0.62(0.32~1.21) | 1.00 | 1.98(1.42~2.77) | 2.19(1.24~3.85) |
| Fetal sex |  |  |  |  |
| Male | 0.50(0.34~0.74) | 1.00 | 1.66(1.28~2.15) | 1.64(0.98~2.72) |
| Female | 0.70(0.45~1.07) | 1.00 | 1.89(1.36~2.61) | 1.98(1.09~3.58) |

^a^ Adjusted for covariates other than subgroup variables. ^b^ Missing data.


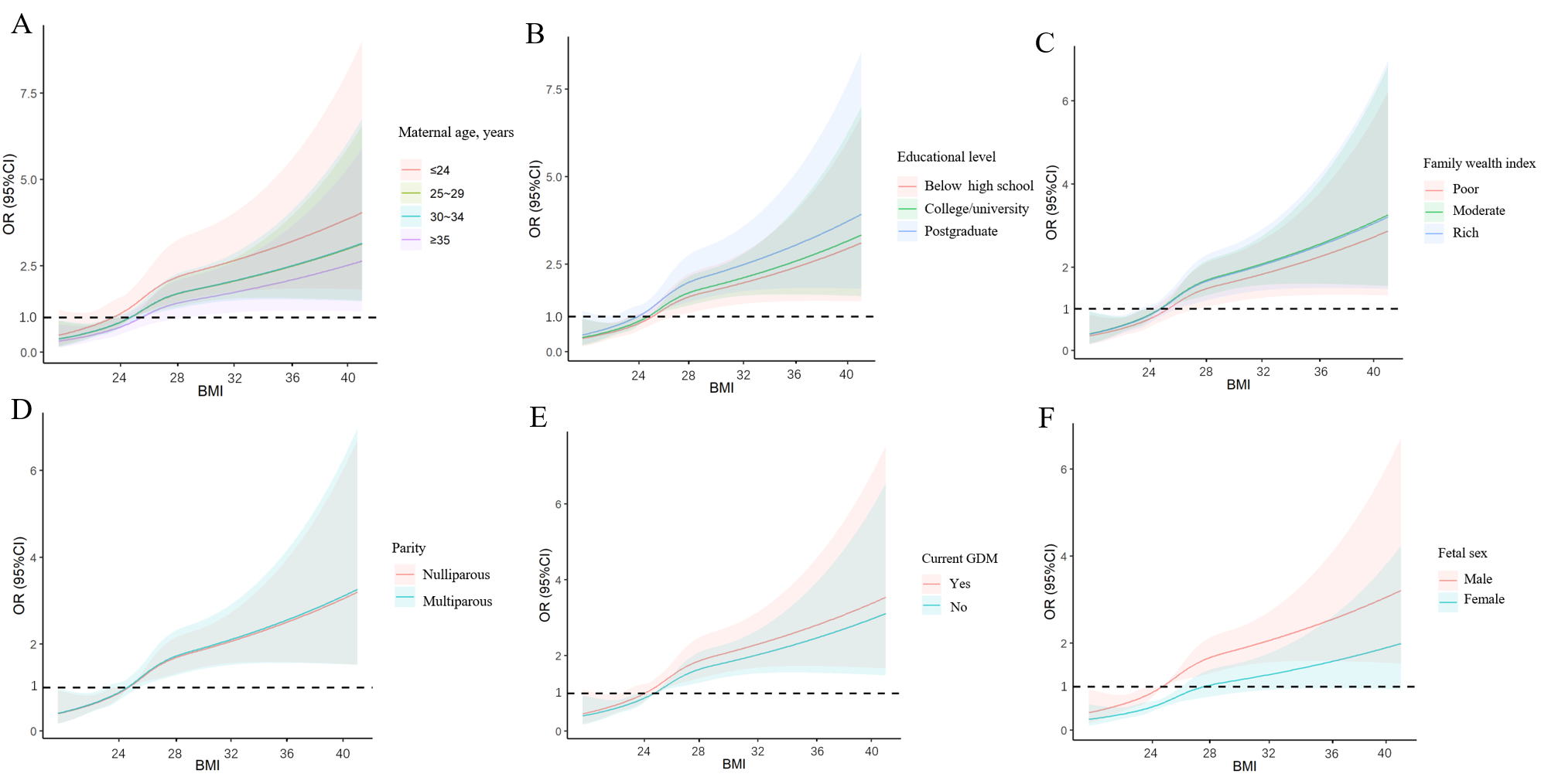


**Supplementary Figure 1** Association of pre-pregnancy BMI with macrosomia: maternal age (A)、maternal education (B)、family wealth index (C)、parity (D)、current GDM (E)、fetal sex (F). Adjusted for covariates other than subgroup variables.


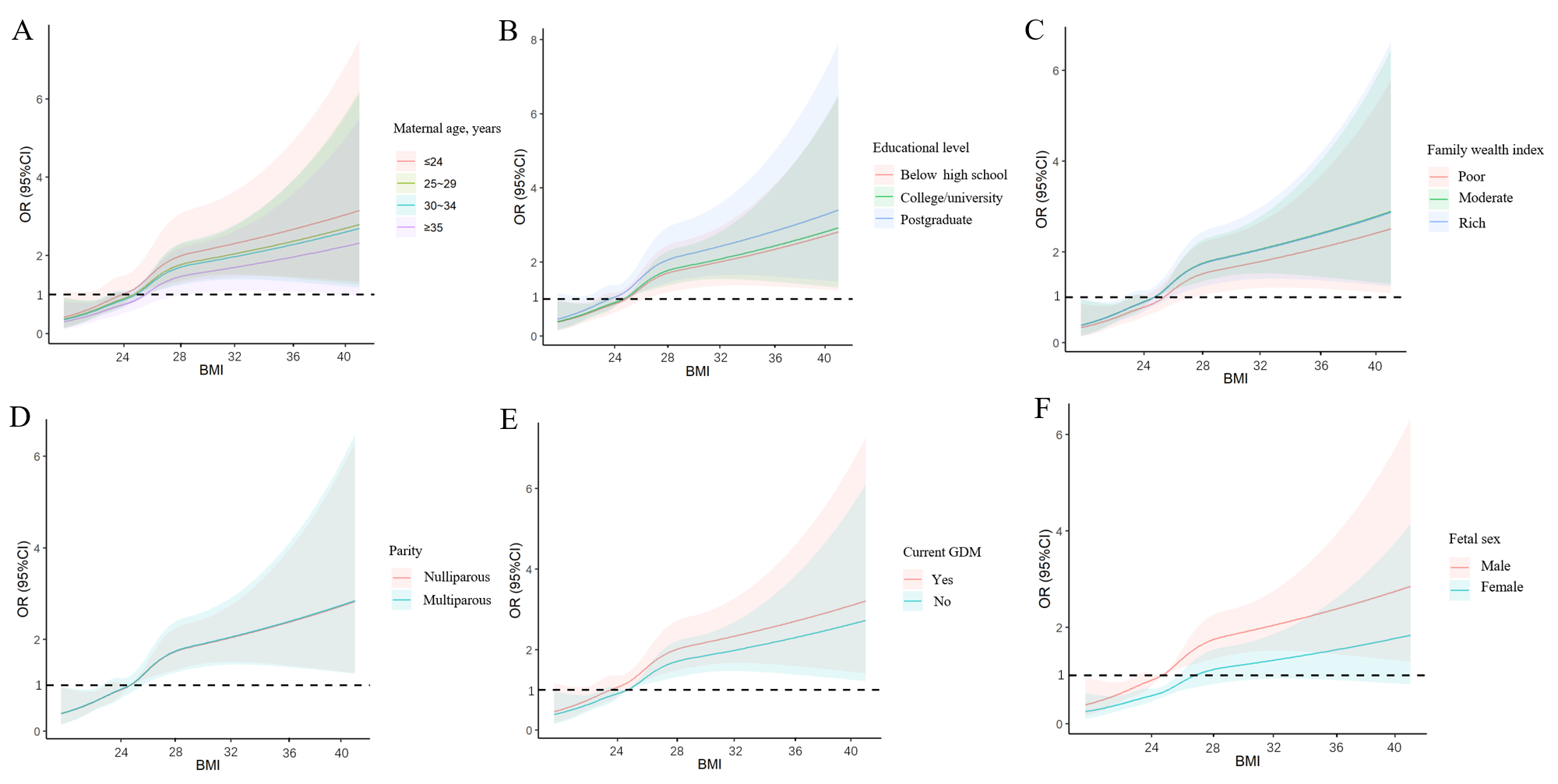


**Supplementary Figure 2** Association of pre-pregnancy BMI with grade 1 macrosomia: maternal age (A)、maternal education (B)、family wealth index (C)、parity (D)、current GDM (E)、fetal sex (F). Adjusted for covariates other than subgroup variables.


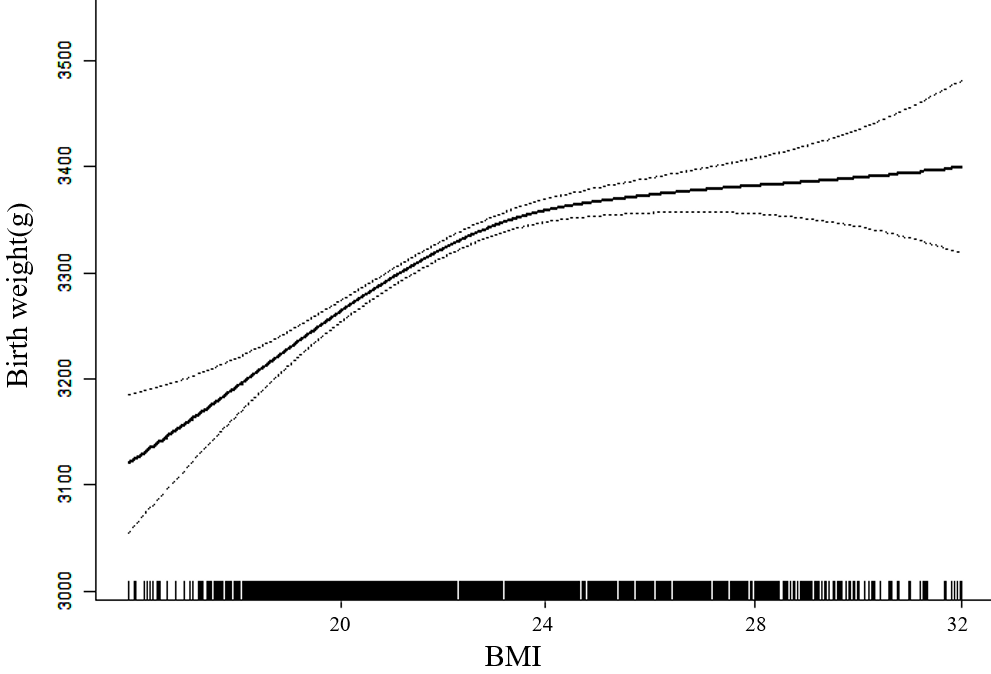


**Supplementary Figure 3** Analysis of the linear relationship between pre-pregnancy BMI and birth weight. Adjusted for maternal age, education level, ethnicity, family financial situation, drinking before or during pregnancy, passive smoke before or during pregnancy, cold/fever before or during pregnancy, folic acid supplementation before or during pregnancy, parity, current GDM, fetal sex.
